# Assessing the robustness of meta-analyses to variability in the frequency and timing of postoperative delirium assessment – a novel methodological approach

**DOI:** 10.1371/journal.pone.0361124

**Published:** 2026-10-22

**Authors:** Maram Khaled, Harsha Shanthanna, Lehana Thabane, James Paul, Anthony Absalom, Sergio Bergese, Federico Bilotta, Douglas Campbell, Matthew Chan, Mark Coburn, David Cowan, Kamal Maheshwari, Edward Mascha, Robert D. Sanders, Daniel I. Sessler, Tim Short, Jessica Spence, Alparslan Turan, Dong-Xin Wang, Maria Wittmann, Maura Marcucci

**Affiliations:** 1 Department of Health Research Methods, Evidence and Impact, McMaster University, Hamilton, Canada; 2 Perioperative and Surgery Research Program, Population Health Research Institute, David Braley Research Institute, Hamilton, Canada; 3 Department of Anesthesia, McMaster University, Hamilton, Canada; 4 Department of Health Research Methods, Evidence and Impact, McMaster University, Hamilton, Canada; 5 Department of Anesthesia, McMaster University, Hamilton, Canada; 6 Department of Health Research Methods, Evidence and Impact, McMaster University, Hamilton, Canada; 7 Department of Anesthesia, McMaster University, Hamilton, Canada; 8 Department of Health Research Methods, Evidence and Impact, McMaster University, Hamilton, Canada; 9 Department of Anesthesiology, University Medical Center Groningen, University of Groningen, Groningen, The Netherlands; 10 Department of Anesthesiology, Neurological Surgery, and Outcomes and Clinical Research in Perioperative Medicine, Stony Brook University, New York, United States of America; 11 Departments of Anesthesiology, Critical Care, and Pain Medicine, Sapienza University of Rome, Rome, Italy; 12 Department of Anesthesia and Perioperative Medicine, Auckland City Hospital, Auckland, New Zealand; 13 Department of Anaesthesia and Intensive Care, The Chinese University of Hong Kong, Hong Kong Special Administration, Hong Kong, China; 14 Department of Anesthesiology and Intensive Care Medicine, University Hospital Bonn, Bonn, Germany; 15 Department of Medicine, McMaster University, Hamilton, Canada; 16 Department of Anesthesiology, Kaiser South Sacramento, California, United States of America; 17 Department of Quantitative Health Sciences, Cleveland Clinic, Ohio, United States of America; 18 Faculty of Medicine and Health, University of Sydney, Sydney, Australia; 19 Center for Outcomes Research and Departments of Anesthesiology and Biostatistics, U T Health, Texas, United States of America; 20 Department of Anesthesia and Perioperative Medicine, Auckland City Hospital, Auckland, New Zealand; 21 Department of Anesthesia, McMaster University, Hamilton, Canada; 22 Department of Health Research Methods, Evidence and Impact, McMaster University, Hamilton, Canada; 23 Perioperative and Surgery Research Program, Population Health Research Institute, David Braley Research Institute, Hamilton, Canada; 24 Center for Outcomes Research and Department of Anesthesiology, Critical Care and Pain Medicine, U T Health, Texas, United States of America; 25 Department of Anesthesiology, Peking University First Hospital, Beijing, China; 26 Clinic and Polyclinic for Anesthesiology and Operative Intensive Care Medicine, University of Bonn, Germany; 27 Clinical Epidemiology and Research Centre (CERC), Department of Biomedical Sciences, Humanitas University and IRCCS Humanitas Research Hospital, Milan, Italy; 28 Department of Health Research Methods, Evidence and Impact, McMaster University, Hamilton, Canada; 29 Perioperative and Surgery Research Program, Population Health Research Institute, David Braley Research Institute, Hamilton, Canada; Menzies School of Health Research: Charles Darwin University, AUSTRALIA

## Abstract

**Background:**

Postoperative delirium (POD) is a fluctuating confusional state occurring early after surgery. Given its fluctuating nature, POD detection is influenced by the frequency and timing of screening. Sparse assessments potentially underestimate the true incidence reported in individual studies, subsequently influencing the weight of studies in meta-analyses, and the results and precision of these meta-analyses.

**Objective:**

To develop a method to evaluate the robustness of meta-analyses to the variation in the frequency and timing of POD assessment across included studies.

**Methods:**

We developed an expert-based scoring system to rate studies according to the confidence that a patient reported not to have POD in the study truly did not, based on the reported timing and frequency of POD assessments. We describe how to use the system to generate hypothetical event rates for studies if the frequency and timing of POD assessment were optimal and how to use these rates in a sensitivity analysis of meta-analyses including these studies. We illustrate the method applying it to two meta-analyses as work examples, one of observational studies and one of randomized controlled trials (RCTs).

**Results:**

In our work examples, the expert-based score for the individual studies ranged from 33–77% for the observational and 22–70% for RCTs meta-analysis. Sensitivity analyses showed a change in the pooled effect size, heterogeneity, and precision compared to the original estimates.

**Conclusion:**

We present a method to evaluate the robustness of a meta-analysis of studies having POD as an outcome, when there is variation across individual studies in the frequency and timing of POD assessment. The confidence score acts as a preliminary index of the robustness of meta-analyses. Through work examples, we showed that the robustness depends on the characteristics of individual studies included.

## 1. Introduction

Postoperative delirium (POD) is an acute fluctuating state of confusion that commonly occurs in older adults after surgery [1,2] and is associated with adverse outcomes [3,4]. The reported incidence ranges from 5–50% [1]. Variation in the observed incidence is presumably mostly explained by risk variation across patient and surgical populations [5,6]. However, an additional factor of variation is how delirium is assessed, for how long, and how often.

Even across studies adopting a systematic assessment with the same validated delirium instrument, the frequency and timing of POD assessment may vary widely [7–9]. The “waxing-and-waning” nature of delirium suggests that just because delirium was not evident at the time of the assessment, one cannot exclude it would have occurred at an earlier or later time. Moreover, although POD usually occurs on the first postoperative day [10,11], confusion can be present up to a week later. Consequently, the frequency and timing of POD assessments critically influence delirium detection, expecting more episodes to be detected with frequent and prolonged assessments [12–14].

For practical reasons, various studies use different assessment approaches. Inadequate assessments diminish apparent incidence and power. When studies using different assessment approaches are combined in a meta-analysis, it is uncertain how and to what extent the difference in frequency and timing of POD assessment affects the results and their interpretation. We proposed a method for evaluating how the results of meta-analyses of comparative studies with POD as an outcome are robust / sensitive to the variation in the frequency, timing and duration of POD assessments across included studies. We present the method and its rationale, then apply it to two existing meta-analyses, one of observational studies and one of randomized controlled trials [RCTs]) as examples to illustrate the application of the method.

## 2. Methods

### 2.1 Overview of the approach

We developed a method to assess the robustness of meta-analyses that include comparative studies in which POD is an outcome assessed with different frequencies, timings and durations. Our approach compares the actual results of the meta-analyses with the results that would be obtained in a hypothetical scenario where all the included studies adopted *optimal* frequency and timing of POD assessment. To calculate the hypothetical results, the results of each included study are to be adjusted according to the extent of deviation of POD assessment in that study from *the optimal* frequency and timing. In the absence of definitive evidence of what constitutes optimal frequency and timing of POD assessment, as a preliminary step, we attempted to define optimal assessment based on expert opinion. We developed a scoring system to rate studies based on *experts’ confidence* that the negative delirium cases reported are truly negative, as per the frequency and timing of POD assessment implemented in that study. The process of score development and how to apply it in running a sensitivity analysis to assess the robustness of a meta-analysis to the variability in the frequency and timing of POD assessment across the included studies are described below.

### 2.2 Development of the expert-based score system

Senior clinicians and researchers from around the world with a track record on POD and related methods were invited to collaborate as expert panelists in this study. We contacted 19 experts, out of whom 17 agreed to collaborate (AA, SB, FB, DC, MC, MC, DC, KM, EM, HS, RDS, DIS, TS, JS, AT, DW, MW) and two did not respond. The panelists were asked to complete a questionnaire created with a focus on the sensitivity of the frequency and timing of POD assessment (and not, for instance, on the validity of the assessment tool used), and how frequency and timing could influence POD detection.

The first and senior authors (MK, MM) pre-identified four key factors related to the frequency and timing of POD assessment which can potentially influence POD detection: 1) number of postoperative days up to which POD is assessed (1–30 days); 2) scheme of POD assessment (everyday *vs*. intermittently); 3) number of times/day POD is assessed; and 4) time of the day POD is assessed (morning *vs*. afternoon *vs*. night). The identification of these factors was informed by the existing literature on the optimal timing of assessment.(12–14)

The full questionnaire is provided in supporting material (S1 File). First, panelists were asked to rank the factors in order of importance in influencing their confidence in POD detection methods. We used the expert ranking to decide the weight of each factor in the calculation of the final score (Table 1), which was determined by the percentage of expert panelists ranking each factor as the most important (rank 1). If the percentage of votes for the first rank were tied, we compared the sum of votes for the first and second ranks for the tied factors.

**Table 1:** Factor weights.

Importance	Factor ranking	Weight (Contribution to the overall score)
Most important	1	35%
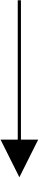	2	30%
	3	20%
Least important	4	15%

Factor weights based on their ranking of importance as assigned by the panelists

After ranking, the panelists were presented, for each factor, with a hypothetical scenario for alternative modalities the factor could take on in a study, assuming the other factors were presumably optimal, and were asked, for each modality, to rate their confidence (0–10 scale, representing 0–100% confidence) that a negative case in a study (no POD) is truly negative, if delirium was assessed according to that modality.

To obtain a confidence scoring system, we used the median of the expert-based confidence ratings assigned by the panelists to each possible factor modality to obtain the score for that modality. For each study, the overall confidence score is the sum of the weighted scores for the individual factors (equation 1.1, 1.2)


$$\begin{array}{cc} Weighted\;factor\;score= & Median\;of\;the\;expert\;based\;confidence\;ratings\;assigned\;to\;the\;factor\;\\ & modality\;used\;in\;the\;study*weight\;of\;the\;factor\;according\;to\;its\;ranking\;\\ & of\;importance \end{array}$$ (1.1)

$$\begin{array}{cc} Total\;confidence\;score= & Weighted\;score\;of\;factor\;\text{1}+\;Weighted\;score\;of\;factor\;\text{2}+\\ & \;Weighted\;score\;of\;factor\;\text{3}+Weighted\;score\;of\;factor\;\text{4} \end{array}$$ (1.2)

Finally, at the end of the questionnaire, panelists were asked if their responses would be different if the study included an additional method of POD assessment (such as chart review). Based on the panelists’ responses to that question we added a pre-determined absolute value of 0.05, 0.1 or 0.15 points to the confidence score if the percentage of panellists responding “yes, confidence increases in all scenarios” was 30–60%, 61–90% or >90% respectively. No points were added if <30% of panelists responded “yes” to that question.

### 2.3 Applying the confidence scoring in sensitivity analyses

To use the score in a sensitivity analysis assessing the robustness of a meta-analysis to the variability in the frequency and timing of POD assessment across the included studies, the first step is to extract, for each individual study, the modalities of the four factors related to the frequency and timing with which POD was assessed. Based on these data, an overall score is calculated for each study as described above. If a study did not report the details of assessment in one or more of the factors, the scenario with the lowest confidence for that factor is to be assumed and its corresponding score is to be used.

In the second step, these individual studies’ scores are used to simulate the hypothetical scenario in which all studies assessed POD with the optimal frequency and timing. Since the score represents the confidence that a given patient reported as without POD in a study was really a negative case, the score can be interpreted as a *negative predictive value* (NPV) score. Therefore, subtracting the score from 1 is equal to the complement of a NPV also called a *false omission rate* (*equation 2*), that is, the proportion of patients, among patients with a negative test, who are in fact diseased. For the current purpose, this can be also seen as the proportion of negative cases in the study that would have been positive if the optimal frequency and timing of POD assessment had been adopted. For example, a score of 0.9 means that out of 10 patients reported as having no POD, 9 are truly negative, and 1 is a POD case that was missed using the methods reported for that study.


$$\begin{array}{c} (\text{1}\;-\;confidence\;score)\;=\frac{Hypothetical\;false\;negative\;POD\;cases}{Reported\;negative\;POD\;cases} \end{array}$$ (2)

Based on *equation 2*, the absolute number of hypothetical false negative cases (which from negative, should be counted as positive POD cases) could be calculated by multiplying the false omission rate (1-confidence score) by the number of non-POD cases in each study. In the hypothetical scenario, the number of POD and non-POD cases, for each study group (intervention/exposure or control), can then be recalculated as per equations 3.1 and 3.2:


$$\begin{array}{cc} Hypothetical\;number\;of\;POD\;cases\;= & \;reported\;number\;of\;POD\;cases\;+\\ & \;hypothetical\;false\;negative\;POD\;cases \end{array}$$ (3.1)

$$\begin{array}{cc} Hypothetical\;number\;of\;non-POD\;cases\;= & \;reported\;number\;of\;non-POD\;cases\;-\\ & \;hypothetical\;false\;negative\;POD\;cases \end{array}$$ (3.2)

As a last step, the hypothetical event rates calculated for each included study can be used in a sensitivity analysis to be compared to original meta-analysis. The extent to which the results differ in terms of effect size, precision, and the statistical heterogeneity of the pooled estimate can provide insight into how robust/sensitive, the results of the meta-analysis are to the variability of POD frequency and timing.

To note, this approach relies on two main assumptions. First, that it is applied to meta-analyses and studies involving cohorts of patients with a fixed, or reasonably similar, underlying POD incidence or pre-test probability. Indeed, a negative predictive value depends not only on the characteristics (i.e., sensitivity) of the assessment tool but also on the prevalence of the disease. Likewise, the experts’ confidence in a negative case might have been different for the same frequency and timing of POD assessment for different underlying POD incidences. By not including the underlying POD incidence in the expert survey, we made the assumption that the experts would rate their confidence thinking of a typical surgical population with a typical POD incidence, and hence, this is the setting to which the elicited confidence score is most applicable. Second, for this primary analysis, we assumed that the missed diagnosis of POD is only related to the non-optimal frequency and timing of assessment and is independent of the effect of intervention/exposure. Therefore, false omission rates (i.e., proportion of missed cases due to non-optimal assessment method) is the same between the intervention/exposure and control groups as per *equation 4*:


$$\begin{array}{c} \frac{Hypothetical\;false\;negative\;POD\;cases\;in\;intervention\;or\;exposure\;group}{Reported\;negative\;POD\;cases\;in\;intervention\;or\;exposure\;group}\;÷\\ \;\frac{Hypothetical\;false\;negative\;POD\;cases\;in\;control\;group}{Reported\;negative\;POD\;cases\;in\;control\;group}=\text{1} \end{array}$$ (4)

### 2.4 Additional analyses

#### 2.4.1 Accounting for potential differential misclassification between study groups.

Our proposed approach assumes that the missed diagnosis of POD is not related to the effect of intervention/exposure or lack thereof. However, this may not always be true, and the proportion of missed events may be different between the intervention/exposure and control groups. This is particularly possible in studies where the effect estimate suggests the intervention/exposure is significantly associated with an increase or decrease in the outcome. In such cases, some of the “false” negative cases attributed to low sensitivity of the assessment may be truly negative, and false negatives may be disproportionate between the two groups. Applying the score equally to the two groups in these cases tends to narrow the gap between the number of events in the two groups and can falsely change the overall pooled effect size or even reverse the direction of the effect, especially in smaller studies with a smaller number of overall events. To address this, we performed the sensitivity analyses also using a second approach that applies the confidence score but that also trusts and accounts for the reported effect size in studies that show significant association between the exposure / intervention and outcome (equation 5)


$$\begin{array}{c} \frac{False\;negatives\;intervention\;or\;exposure\;group}{Reported\;negatives\;in\;intervention\;or\;exposure\;group}\;÷\;\frac{False\;negatives\;in\;control\;group}{Reported\;negatives\;in\;control\;group}=Reported\;effect\;estimate \end{array}$$ (5)

According to this equation, we assume that if the intervention or exposure is associated with the outcome (i.e., the effect estimate is statistically significant), the hypothetical proportion of false negatives in the intervention/exposure group is greater (if effect estimate >1) or less (if effect estimate <1) than that in the control group by a value which is explained by the effect estimate. We applied this adjustment to calculate the false negatives, and subsequently hypothetical event rates, in only one of the intervention / exposure or control group whereas the false negatives in the other group were calculated using only equations *1-3*. Depending on the direction of the association, we decided which group to apply the adjustment in to avoid overinflation of event rates beyond the total sample size of the study. For studies showing no significant association between the intervention/exposure and outcome, we assume that the proportion of false negatives to reported negatives is equal in the intervention/exposure and control groups *(*equation *4)*.

#### 2.4.2 Evaluating the sensitivity of the proposed approach to the expert-based weights.

In the proposed approach, we assigned weights to each factor based on the expert-based rank and pre-defining the weights associated with each rank (35%, 30%, 20% and 15%). To evaluate the sensitivity of our approach to the expert-based weights, we conducted an additional analysis using equal weights (25%) for all factors (i.e., assuming all factors are equally important).

#### 2.4.3. Power transformation analysis.

We conducted additional analyses using power transformation estimating the hypothetical false omission rate by (1-confidence score)^k^, where our original linear mapping corresponds to k = 1. We used values for k to represent a conservative (k = 2 – estimated false omission rate declines more steeply as confidence increases), or aggressive (k = 0.5 – elevated false omission rates persist even at moderate to high confidence scores) scenarios, repeated the analyses under each scenario (results in supplementary material S5 File).

## 3. Results

### 3.1 The expert-based confidence score

Panelists completed the questionnaire between April and May 2023. Fig 1 shows the distribution of votes for all factors across ranks 1–4 and the ranking of the four factors determined by the percentage of expert panelists voting each factor as the most important (rank 1). Fig 2 shows the median confidence scores for the modalities each factor could take on. The number of times/day delirium was assessed was ranked as the top factor influencing the panelists’ confidence, followed by the number of postoperative days up to which POD was assessed. Median confidence scores increased from one to three assessments/day; and with higher numbers of postoperative days, with a plateau after day seven. The use of additional POD assessment methods such as chart review was valued as a factor increasing confidence by 76.5% of panelists. As per our a priori criteria this resulted in a bonus 0.1 points added to the overall score if an additional POD assessment method was reportedly used. Based on the above, we calculated the attainable overall confidence score for any study could range from 0.215–0.925.

**Fig 1:**
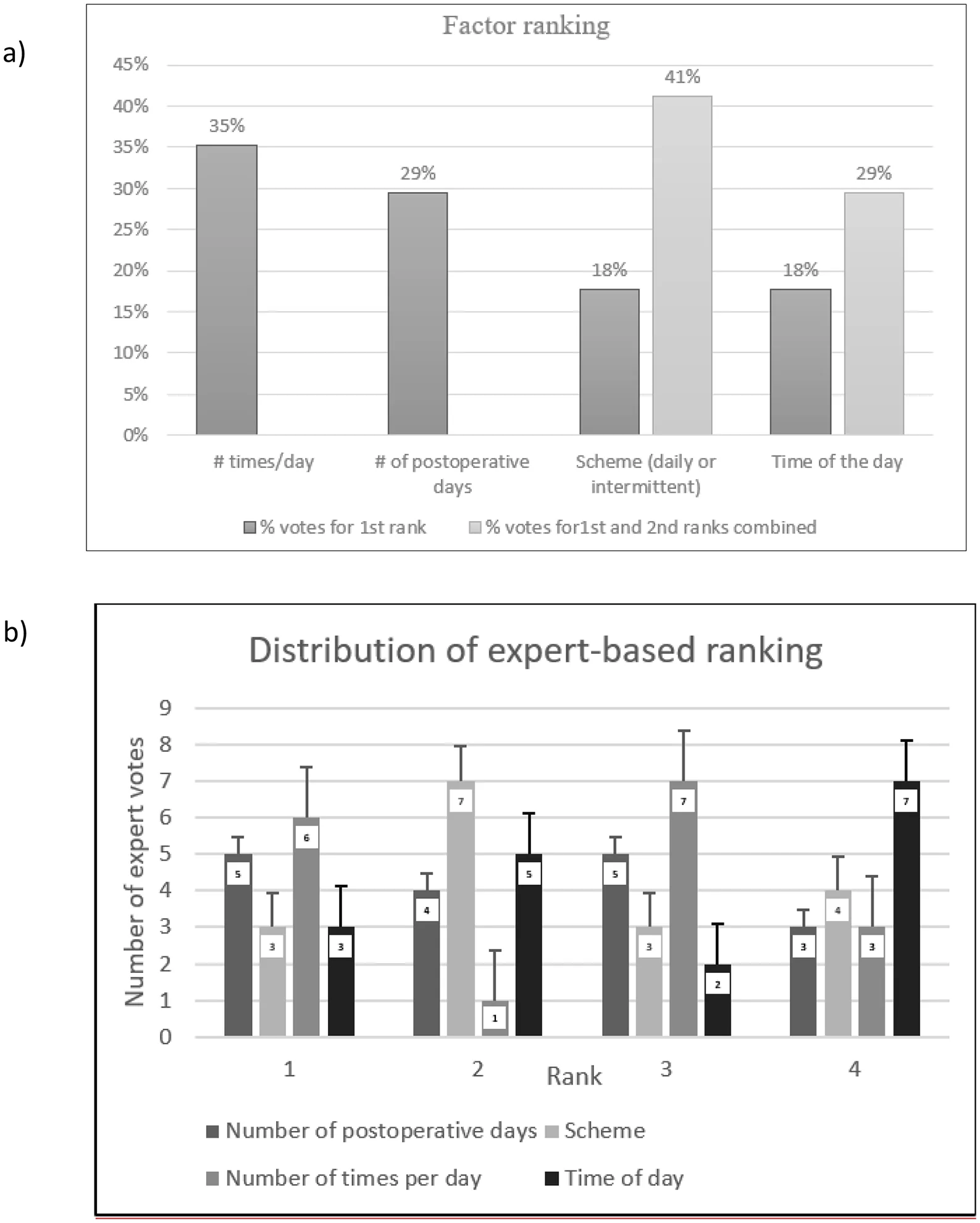
Factor ranking based on their influence on the confidence in reported no-POD events. Fig 1(a) shows the percentage of panelists voting each factor as the first rank. For factors with a tied percentages of votes as first rank, the figure additionally shows the percentages of votes as second rank. Fig 1(b) shows the distribution of votes for all factors across ranks 1 to 4.

**Fig 2:**
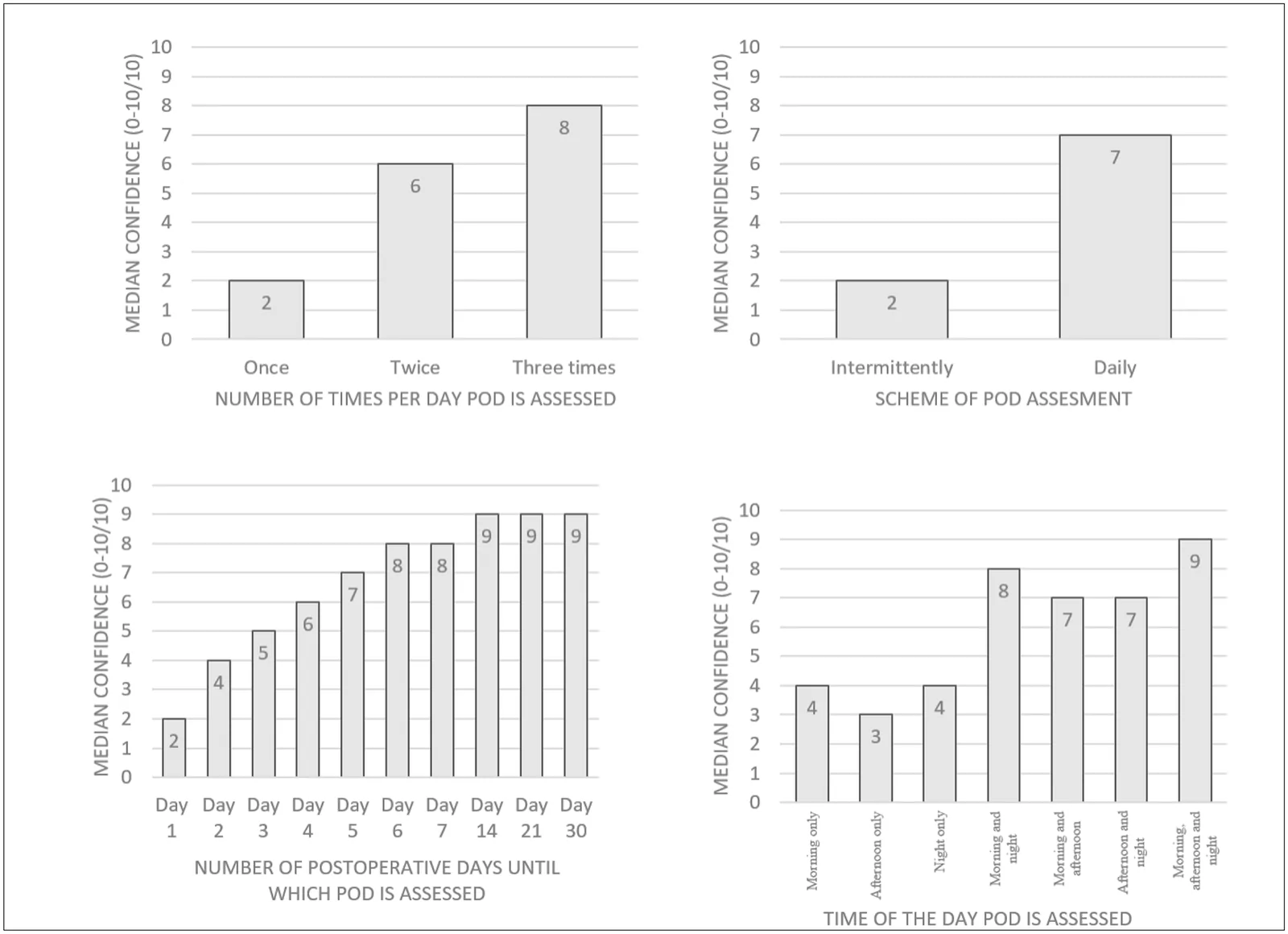
Median confidence scores for each scenario of POD assessment. The graphs represent the median confidence scores for each modality of each of the four factors related to the frequency and timing of POD assessment.

### 3.2 Work examples

#### 3.2.1 Fully worked calculation in an individual study.

We illustrate our method using an example from a randomly selected published study to demonstrate one fully worked calculation from reported to hypothetical counts (Table 2). Ali et al. [15], is an observational study evaluating the incidence and risk factors of delirium in surgical ICU. For this example, we will use postsurgical pain as the exposure.

**Table 2:** Step-by-step demonstration of a full worked example for one study.

Step 1: Calculating the confidence score			
1. Extract data regarding the 4 factors below describing the methods of POD assessment from each study			
Factor 1 – Number of days: 5 days			
Factor 2 – Number of times / day: 3 times			
Factor 3 – Scheme: Daily			
Factor 4 – Time of day: Morning, afternoon and night			
2. Calculate the weighted factor scores ◊ (*equation 1.1*) Median of the expert-based confidence ratings assigned to the factor modality used in the study*weight of the factor according to its ranking of importance			
	**a.** Corresponding median score (Fig 2)	**b.** Weight (Table 1 and Fig 1a)	Weighted score (a*b)
Factor 1	0.7	0.30	0.21
Factor 2	0.8	0.35	0.28
Factor 3	0.7	0.20	0.14
Factor 4	0.9	0.15	0.135
3. Bonus for additional method of assessment? No			
4. Calculate total confidence score ◊ (*equation 1.2*) Weighted score of factor 1 + Weighted score of factor 2 + Weighted score of factor 3 + Weighted score of factor 4			
Total confidence score = 0.21 + 0.28 + 0.14 + 0.135 = 0.765			
**Step 2: Calculating Hypothetical event rates**			
1. Extract the total number of participants per group and the number of events reported in each of the intervention / exposure and the control group			
	Total n	Events (reported)	Non-events (reported negative POD cases = Total n-events)
Exposure	19	11	8
Control	68	8	60
2. Calculate the hypothetical false negative POD cases ◊ (*equation 2*) (1 – confidence score) = (False negative POD cases)**/**(Reported negative POD cases)			
Exposure Control	False negative POD cases = (1–0.765) * 8 = 1.88 ◊ round to 2 False negative POD cases = (1–0.765) * 60 = 14.1◊ round to 14		
3. Calculate hypothetical event rates ◊ (*equation 3.1*) Hypothetical number of POD cases = reported number of POD cases + hypothetical false negative POD cases			
Exposure Control	Hypothetical number of POD cases = 11 + 2 = 13 Hypothetical number of POD cases = 8 + 14 = 22		

To further facilitate the application of our method, we prepared an interactive step-by-step tool (Supporting material S2 File), which uses data extracted from individual studies to calculate confidence scores and hypothetical event rates which can be used to conduct sensitivity analyses.

#### 3.2.2 Work examples in two meta-analyses.

We illustrate the proposed sensitivity analysis approach, applying it to two published meta-analyses as work examples. The first is a meta-analysis conducted by our team evaluating the association between moderate-severe vs. no or mild postoperative pain and the incidence of POD in six observational studies [7]. The second is a meta-analysis of the effect of perioperative use of benzodiazepines on the incidence of POD including seven RCTs (data shared by courtesy of the authors) [8]. Both meta-analyses used random-effects models, and we followed the same for our sensitivity analyses.

Table 3 shows the confidence score obtained for each study included in the two meta-analyses [7,8], based on their reported methods of POD assessment. Supporting material (S3 File) show the details of score application to the included individual studies to obtain the hypothetical event rates, according to *equations 2* and *3,* assuming optimal POD assessment scenarios.

**Table 3:** Expert-based confidence scores for each factor related to the frequency and timing of POD assessment and overall, for the studies included in the two work examples.

a) Studies included in the meta-analysis of observational studies													
Study	# of postoperative days up to which POD is assessed			# of times/day POD was assessed			Scheme			Time of day POD is assessed			Overall confidence score
	Weight: 0.3			Weight: 0.35			Weight: 0.2			Weight: 0.15			
	Data from study	Corresp-onding median	Weighted score	Data from study	Corresp-onding median	Weighted score	Data from study	Corresp-onding median	Weighted score	Data from study	Corresp-onding median	Weighted score	
Morrison 2003	**5**	0.7	0.21	**Not** **reported**	0.2	0.07	**Daily**	0.7	0.14	**Not** **reported**	0.3	0.045	**0.465**
Vaurio 2006	**2**	0.4	0.12	**1**	0.2	0.07	**Daily**	0.7	0.14	**Morning only**	0.4	0.06	**0.39**
Lin 2016	**3**	0.5	0.15	**2**	0.6	0.21	**Daily**	0.7	0.14	**Morning and night**	0.8	0.12	**0.62**
Alvarez-Bastidas 2018	**1**	0.2	0.06	**1**	0.2	0.07	**Daily**	0.7	0.14	**Morning only**	0.4	0.06	**0.33**
Iamaroon 2020	**7**	0.8	0.24	**1**	0.2	0.07	**Daily**	0.7	0.14	**Morning only**	0.4	0.06	**0.51**
Ali 2021	**5**	0.7	0.21	**3**	0.8	0.28	**Daily**	0.7	0.14	**Morning, afternoon and night**	0.9	0.135	**0.765**
**b) Studies included in the meta-analysis of RCTs**													
**Study**	**# of postoperative days up to which POD is assessed**			**# of times/day POD was assessed**			**Scheme**			**Time of day POD is assessed**			**Overall confidence score**
	**Weight: 0.3**			**Weight: 0.35**			**Weight: 0.2**			**Weight: 0.15**			
	**Data from study**	**Corresp-onding median**	**Weighted score**	**Data from study**	**Corresp-onding median**	**Weighted score**	**Data from study**	**Corresp-onding median**	**Weighted score**	**Data from study**	**Correspo-nding median**	**Weighted score**	
Aizawa 2002	**7**	0.8	0.24	**2**	0.6	0.21	**Daily**	0.7	0.14	**Morning and afternoon**	0.7	0.105	**0.695**
He 2018	**5**	0.7	0.21	**1**	0.2	0.07	**Daily**	0.7	0.14	**Not** **reported**	0.3	0.045	**0.465**
Imran-ul-Hassan 2021	**3**	0.5	0.15	**1**	0.2	0.07	**Daily**	0.7	0.14	**Not** **reported**	0.3	0.045	**0.405**
Silva-Jr 2019	**1**	0.2	0.06	**1**	0.2	0.07	**Not** **reported**	0.2	0.04	**Not** **reported**	0.3	0.045	**0.215**
Spence 2020	**2**	0.4	0.12	**2**	0.6	0.21	**Daily**	0.7	0.14	**Morning and night**	0.8	0.12	**0.59**
Sultan 2010	**3**	0.5	0.15	**1**	0.2	0.07	**Daily**	0.7	0.14	**Not** **reported**	0.3	0.045	**0.405**
Yu 2017	**3**	0.5	0.15	**1**	0.2	0.07	**Not** **reported**	0.2	0.04	**Not** **reported**	0.3	0.045	**0.305**

#### 3.2.3 Work example 1 - Meta-analysis of observational studies.

The original meta-analysis showed a significant association between postoperative pain and POD (odds ratio [OR] 3.92, 95% confidence interval [CI]: 1.98–7.73; heterogeneity: *I*^2^ = 62% [χ^2^ = 13.15, p = 0.02]) (Fig 3a). In the sensitivity analysis based on an optimal POD assessment scenario (Fig 3b), the point estimates of individual studies shifted towards the line of no effect, with narrower CIs, (overall OR of 1.49, 95% CI: 1.01–2.22; heterogeneity: *I*^2^ = 33% [*X*^2^ = 7.49, p = 0.19]). Compared to the original meta-analysis, the sensitivity analysis showed a smaller overall effect size, narrower pooled CI, and smaller non-significant degree of heterogeneity.

**Fig 3:**
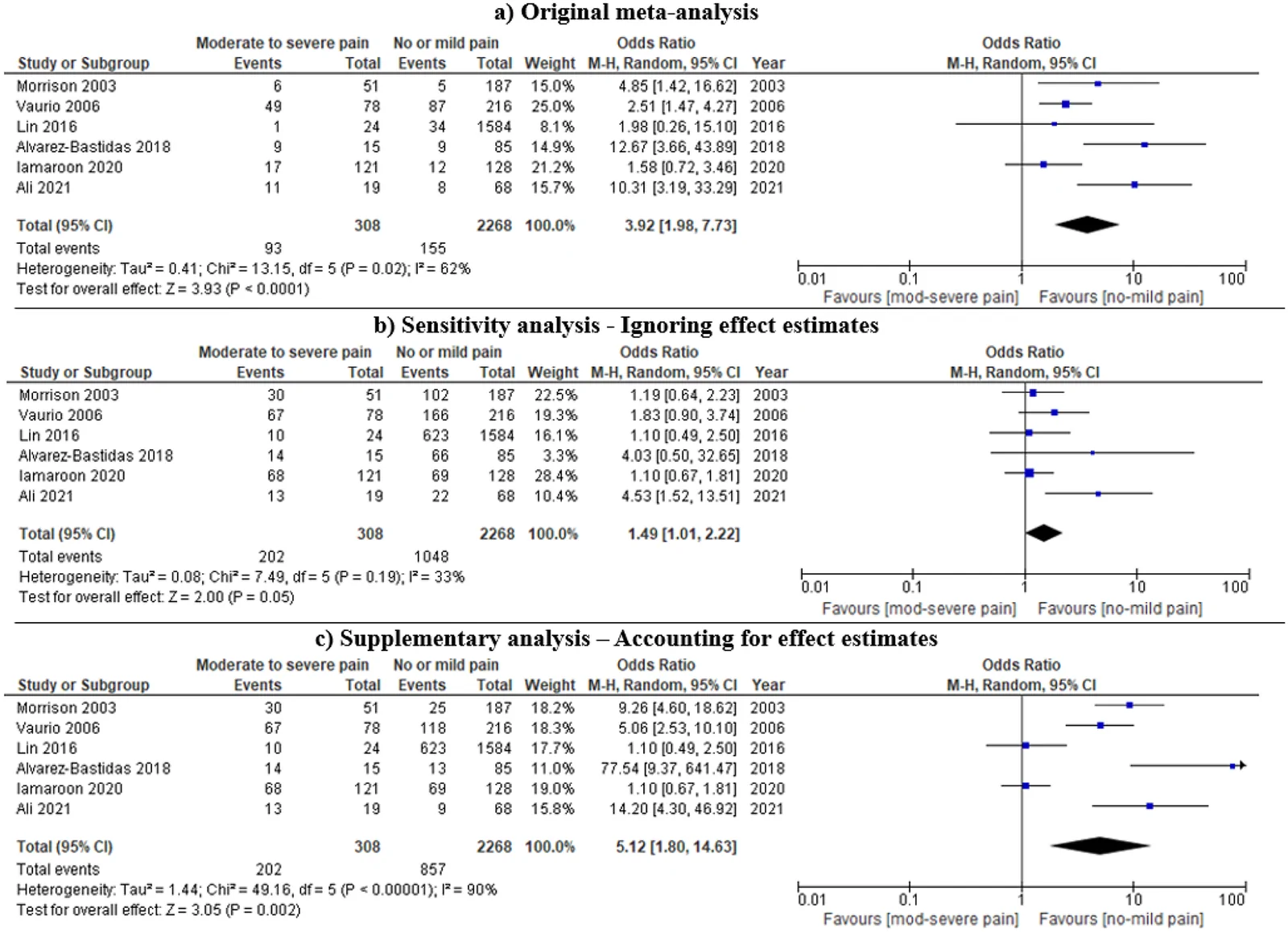
First work example: Meta-analysis of observational studies. Sensitivity analysis of the meta-analysis of observational studies assessing the association between postoperative pain and the incidence of POD. a) The top panel shows a random effects meta-analysis using the event rates reported in individual studies, b) the middle panel shows the sensitivity analysis using the hypothetical event rates based on the confidence scores assigned to individual studies, c) the bottom panel shows the meta-analysis using the hypothetical event rates calculated accounting for the statistically significant effect estimates when applying the score to the exposure and the control group.

#### 3.2.4 Work example 2 - Meta-analysis of RCTs.

In the original meta-analysis, one of the seven included studies reportedly showed zero events in either the intervention and the control groups [16], and therefore its effect size could not be estimated. In the sensitivity analysis, all seven studies provided effect sizes that could be pooled. The original meta-analysis showed no significant association between perioperative use of benzodiazepines and POD (OR:1.19, 95% CI:0.89–1.57; heterogeneity: *I*^*2*^ = 77% [*X^2^* = 21.89, p = 0.0005]) (Fig 4a).(8) Similarly, the sensitivity analysis found no significant association between benzodiazepine use and POD (OR: 1.01, 95% CI: 0.81–1.27; heterogeneity: *I*^*2*^ = 0% [*X^2^* = 4.59, p = 0.60]) (Fig 4b). The pooled CI was narrower in the sensitivity analysis as compared to the original meta-analysis with a lower non-significant degree of heterogeneity. Point estimates of individual studies also shifted towards the line of no effect with narrower confidence intervals.

**Fig 4:**
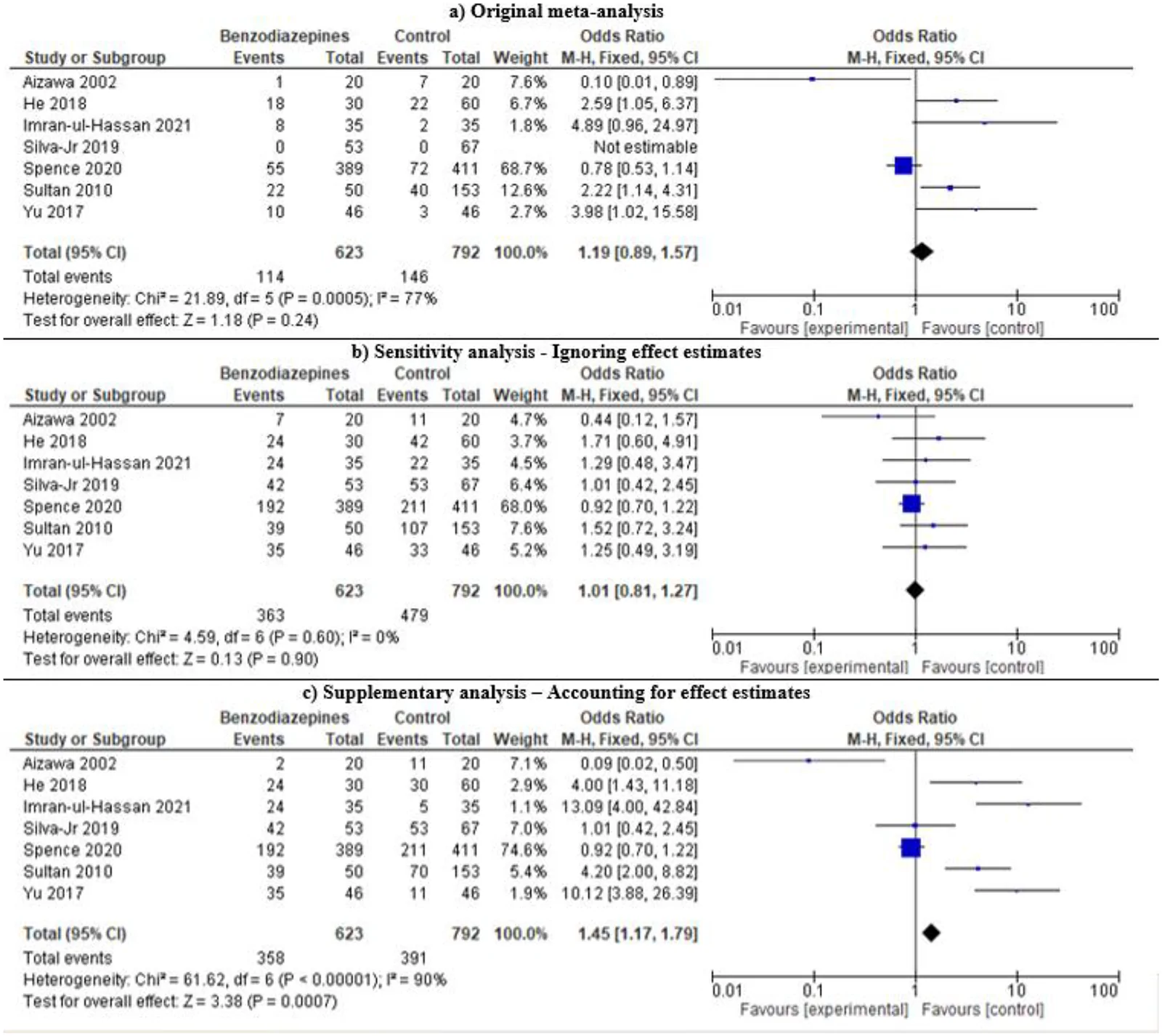
Second work example: Meta-analysis of RCTs. Sensitivity analysis of the meta-analysis of RCTs assessing the effect of perioperative benzodiazepines on the incidence of POD. a) The top panel shows a random effects meta-analysis using the event rates reported in individual studies, b) the middle panel shows meta-analyses using the hypothetical event rates based on the confidence scores assigned to individual studies, c) the bottom panel shows the meta-analysis using the hypothetical event rates calculated accounting for the statistically significant effect estimates when applying the score to the exposure and the control group.

### 3.3 Additional analyses

#### 3.3.1 Accounting for potential differential misclassification between study groups.

*3.3.1.1 Work example 1 – Meta-analysis of observational studies:* Out of six studies in the original meta-analysis, four studies showed a significant association between postoperative pain and POD (OR>1, p < 0.05). As a supplementary analysis, repeating the sensitivity analysis trusting the original statistically significant effect estimates, hypothetical event rates for the control group in these four studies were calculated using equations *1-4*, whereas the event rates in the remaining two studies without statistically significant effect estimates were calculated using equations *a-c* as described above. The supplementary sensitivity analysis (Fig 3c) revealed a larger effect and wider confidence interval than the original meta-analysis, with a higher degree of heterogeneity (OR: 5.12, 95%CI: 1.80–14.63, I^2^:90% [*X*^2^ = 49.16, p < 0.00001]). The effect size of each of the four studies showing significant association in the original meta-analysis was larger in the sensitivity analysis, and the confidence intervals of three of the four studies were wider.

*3.3.1.2 Work example 2 – Meta-analysis of RCTs:* In this work example, as a supplementary analysis, the sensitivity analysis was repeated trusting the original statistically significant effect estimates in five studies using *equations 1-3,* and *equation 5*. In the original meta-analysis of RCTs, four out of seven included studies showed a significant increase in the risk of POD with the use of perioperative benzodiazepines (OR>1, p < 0.05). In the supplementary analysis of these studies, after calculating hypothetical event rates in the intervention group *(equations 1-4)*, the event rates for the control group were calculated relative to those in the intervention group using *equation 5.* One study showed the risk of POD was higher in the control group than in the intervention group. For that study the event rates in the control group were calculated first *(equations 1-4)* then the intervention group event rates were calculated using equation *5.* The supplementary analysis showed a significant increase in the risk of POD with the use of perioperative benzodiazepines, with a higher degree of heterogeneity (OR: 1.45, 95% CI: 1.17–1.79, *I*^*2*^:90% [*X*^2^ = 61.62, p < 0.00001]) (Fig 4c).

#### 3.3.2 Evaluating the sensitivity of the method to the expert-based weights.

Repeating the analyses applying equal weights to all factors (i.e., 0.25), we observed a change in individual study confidence scores ranging from −0.04 to 0.01 (median = −0.02). The results of the sensitivity meta-analyses remained consistent with our primary sensitivity analysis using factor weights based on expert-based ranking (supplementary material S4 File).

## 4. Discussion

We propose an approach for a sensitivity analysis that assesses the robustness or sensitivity of meta-analyses of studies evaluating associations between an exposure/intervention and POD to the variation in the frequency and timing of POD assessment across the included studies. The approach is based on the comparison between the actual results of the meta-analysis, and the results that one would obtain if the included studies evaluated POD implementing optimal frequency and timing of assessment (provided a validated POD assessment tool was used). Each study is assigned a preliminarily developed score which measures the confidence that experts would have for the negative POD cases in the study being truly negative. Based on the score, a number of POD cases that were possibly missed by the study but that would have been found in the ideal scenario are moved from the negative POD to the positive POD cases, in both groups (exposure and non-exposure, or intervention and control groups). Therefore, the lower the confidence scores in individual studies, the greater is the number of the added POD cases, proportionally to the number of reported negative cases. We expect the addition to influence the results of the original meta-analysis, with different point estimate and precision of the pooled effect size, and in degree of statistical heterogeneity.

We used work examples to show how meta-analyses can be robust or sensitive to the variation in the frequency and timing of POD assessment. In both examples, we observed a decrease in the effect size (i.e., a shift towards the null effect of the point estimate), higher precision, and lower heterogeneity. These are expected directions of the impact of simply adding positive POD cases, by a factor determined by the confidence score, proportionally to the original number of negative cases in both groups, without correction for the possible differential effect of the intervention vs. control on the outcome. If groups are of similar size, the percent increase in event rate will be higher in the group in which a lower event rate was originally found. However, it is the extent of these differences, and how these would impact the conclusions that one would draw from the meta-analysis, that gives a sense of the vulnerability of the meta-analysis to the variability of frequency and timing of POD assessment across the included studies. Even if meta-analyses including studies with low confidence scores are more prone to having different results in the sensitivity vs the original analysis, the impact is not fully predictable based on the confidence score, rather it is driven by the combination between the confidence score of each study and its number of events, sample size, and sample size distribution between the intervention/exposure and control groups. For instance, although the mean confidence score of the included studies in the meta-analysis of observational study (0.51) was higher than that of RCTs (0.46), the vulnerability of the former seemed greater than the latter. This differential effect of increasing positive POD cases even among the non-exposed could be partially explained by unbalanced sample size distribution between the intervention/exposure group and the control group in the observational studies. Whereas in RCTs, the sample size is usually balanced between the intervention and control groups, in observational studies this is not always the case as there is no control over assigning exposure.

In both work examples, the results of the sensitivity analyses vary when we applied the version of the method trusting and accounting for the reported effect estimate in the individual studies. In the meta-analysis of observational studies, the sensitivity analysis using this version of the method produced a large increase in the effect size with lower precision. Notably, in this example, studies showing a significant association between pain and POD reported large effect sizes. Accounting for these large effects widened the gap between the number of events in the exposure and the control groups, contributing to the larger overall effect. At the same time, studies showing no significant association had relatively smaller effect sizes in the original meta-analysis. This discrepancy between the two sets of studies contributed to the higher heterogeneity we observed. In the meta-analysis of RCTs, this version of the method also produced an increase in effect size, with a pooled estimate that became significant, higher heterogeneity and similar precision to the original meta-analysis. However, the magnitude of increase in effect size was smaller than that observed in the observational studies meta-analysis. Notably, in the RCTs work example, although several small studies suggested an association between benzodiazepines and POD, the largest study, which also had the second highest confidence score, showed no significant association. This suggests that when this version of the proposed method is used, the robustness of the meta-analysis to the variation in frequency and timing of POD assessment across studies will more evidently depend also on the sample size of included studies and the original effect sizes.

This is the first study to propose an approach for evaluating the robustness of meta-analyses to the variability in the timing and frequency of POD assessments across the included studies. In systematic reviews, comparative effectiveness conclusions can vary with methodological variations such as outcome definition, outcome ascertainment and trial composition [17,18]. This is particularly relevant for POD, not only because of the large variability of outcome ascertainment methods across the literature, but also because of the impact this has on delirium detection. As an additional deliverable we generated an expert-based score to rate the appropriateness of frequency and timing of POD assessment in a study. We acknowledge that the score requires further validation before being used as a tool to assess the quality of studies with delirium as an outcome. Nonetheless, it could be used to guide the design of such studies. We found only three studies focusing on the optimal timing and frequency for delirium detection [12–14], none of which implemented an experimental design to compare different POD assessment modalities. One prospective cohort study (1097 patients) concluded that POD should be assessed on the first five postoperative days, with the highest incidence on the first two postoperative days [14]. Another prospective study found that twice daily assessments for four postoperative days are likely to detect nearly all cases of delirium as compared to twice daily assessments for five days, and that POD was detected more in the morning than the evening [13]. A small study (15 patients) found no significant difference in the incidence of POD based on the time of day delirium was assessed [12]. In addition, in a 2013 systematic review, Zhang et. al developed a score to assess the quality of studies based on the frequency and duration of POD assessment, however, the score development and choice of factors were not described [9].

Our study has several strengths. We generated an expert-based score, examining several factors which are commonly variable between studies, to rate how optimal POD assessment is in a study if a valid tool is used. It should be noted that our scoring system is a preliminary, expert-derived, hypothesis-generating framework rather than a validated quality assessment tool and should be applied as such. If the score is used in future meta-analyses, it should be considered a preliminary index of the robustness of the results to the variability in POD assessment methods across included studies, rather than a definitive correction factor. We are currently designing a study to validate the score using large study-level data. We have shown that meta-analyses can be impacted by the variation in POD assessment and that their robustness to these variations are affected by several factors, including study design, confidence scores of included studies, effect size and sample size of individual studies. We assessed the rigor of our methods and stability of our scoring system under different conditions.

Our proposed approach has limitations. First, our conceptualization of 1-the confidence score is an estimate of false omission rate, which is a function of a test’s sensitivity, specificity and the prevalence or incidence of the disease. Therefore, by eliciting the experts’ confidence without specifying different scenarios with different true POD incidences, we cannot exclude that the experts’ confidence would have varied, like any NPV, based on different POD incidence. Caution should then be taken in applying our method to meta-analyses in which the studies appear to include very diverse surgical populations in which to expect a quite different pre-test probability of POD. We focused on the effect of the variation in frequency and timing of POD assessment that is due to random error. In fact, POD assessment could be systematically missed in a certain direction due to reasons that are related to the intervention/exposure. For example, assessor may choose to omit delirium assessments when patients have intense pain, generating a systematic difference in the frequency of POD assessment between the exposed and non-exposed groups. We assumed that the proportion of hypothetical false negatives to reported negatives is the same between intervention/exposure and control groups. Although this approach seems fair, the underlying assumption may not be true. Our supplementary analysis which trusts a potential effect of the intervention on the outcome, as demonstrated by a statistically significant effect estimate, attempts to address this issue of differential misclassification. However, that approach also has limitations. First, it relies on categorizing studies based on statistical significance, which could treat studies with nearly identical effect estimates differently, and second, setting the false-omission-rate ratio as equal to the reported effect estimate partially incorporates the observed effect into the correction, which contributes to the larger estimates and heterogeneity seen in the supplementary analyses. Another limitation of our study is that, in the proposed approach, the assessment of the robustness or vulnerability of the meta-analysis of interest is based on a qualitative comparison of the hypothetical *vs*. original scenarios, without any formal quantitative comparison which would be still limited if not inappropriate [19]. Finally, relying on the opinion of our expert panel *per se* can be considered a limitation. The lack of evidence from the literature regarding optimal frequency and timing of POD assessment, as noted, limited our sources for creating the score. The questionnaire we used is also potentially subject to different types of response bias such as end-aversion and neutral response bias. Indeed, none of the panelists scored any of the factor modalities a perfect score (10/10). Subsequently, the highest attainable score that could be assigned to a study is < 1.

## 5. Conclusion

We propose an approach to assess the robustness of meta-analyses to the variation in the timing and frequency of POD assessment in the included studies. Our work examples suggest that the variation in the frequency and timing of POD assessment between individual studies can have an impact on the precision and heterogeneity of the results (and thus on the certainty of evidence) of meta-analyses. Our approach can be expanded to account for a non-optimal frequency or timing of POD assessment as a source of bias. Further research should be also directed towards determining the optimal frequency and timing of POD assessment, also considering efficiency and feasibility.

## Supporting information

S1 File Details of the survey questionnaire for expert panelists. (DOCX)

S2 File Interactive tool. (XLSX)

S3 File Demonstrating calculation of the hypothetical number of events. (DOCX)

S4 File Additional analysis – Evaluating the sensitivity of the method to expert-based weights. (DOCX)

S5 File Additional analysis – Power transformation analysis. (DOCX)
