# Supplementary material for: Assessing the robustness of meta-analyses to variability in the frequency and timing of postoperative delirium assessment – a novel methodological approach
Source: PLoS One. 2026 Oct 22;21(10):e0361124. doi: 10.1371/journal.pone.0361124 (PMC13690229; doi:10.1371/journal.pone.0361124)
Supplement: Supplementary file 2 [file pone.0361124.s003.docx]

Supporting Material

**S3:** Calculation of the hypothetical number of events

Score application to individual studies included in the meta-analyses and calculation of the hypothetical number of events in the exposure/intervention and the control groups

| **Studies included in the meta-analysis of observational studies** | | | | | | | | | | | |
| --- | --- | --- | --- | --- | --- | --- | --- | --- | --- | --- | --- |
|  | **Overall Confidence** | | **Group** | **Reported # of events** | **Reported # of non-events** | **Hypothetical # of events** | **Hypothetical # of non-events** | **Reported effect estimate** | **Statistically significant?** | **Hypothetical # of events** | **Hypothetical # of non-events** |
|  |  |  |  |  |  | **Ignoring effect estimate** | |  |  | **Accounting for effect estimate** | |
| **Iamaroon 2020** | **0.51** | | **Exposure** | 17 | 104 | 68 | 53 | **1.58** | **No** | 68 | 53 |
|  |  |  | **Control** | 12 | 116 | 69 | 59 |  |  | 69 | 59 |
| **Vaurio 2006** | **0.39** | | **Exposure** | 49 | 29 | 67 | 11 | **2.51** | **Yes** | 67 | 11 |
|  |  |  | **Control** | 87 | 129 | 166 | 50 |  |  | 118 | 98 |
| **Morrison 2003** | **0.465** | | **Exposure** | 6 | 45 | 30 | 21 | **4.85** | **Yes** | 30 | 21 |
|  |  |  | **Control** | 5 | 182 | 102 | 85 |  |  | 25 | 162 |
| **Ali 2021** | **0.765** | | **Exposure** | 11 | 8 | 13 | 6 | **10.31** | **Yes** | 13 | 6 |
|  |  |  | **Control** | 8 | 60 | 22 | 46 |  |  | 9 | 59 |
| **Alvarez-Bastidas 2018** | **0.33** | | **Exposure** | 9 | 6 | 14 | 2 | **12.67** | **Yes** | 14 | 1 |
|  |  |  | **Control** | 9 | 76 | 66 | 19 |  |  | 13 | 72 |
| **Lin 2016** | **0.62** | | **Exposure** | 1 | 23 | 10 | 14 | **1.98** | **No** | 10 | 14 |
|  |  |  | **Control** | 34 | 1550 | 623 | 961 |  |  | 623 | 961 |
|  | | | | | | | | | | | |
| **Studies included in the meta-analysis of observational studies** | | | | | | | | | | | |
| **Aizawa 2002** | **0.695** | **Intervention** | | 1 | 19 | 7 | 13 | **0.1** | **Yes** | 2 | 18 |
|  |  | **Control** | | 7 | 13 | 11 | 9 |  |  | 11 | 9 |
| **He 2018** | **0.465** | **Intervention** | | 18 | 12 | 24 | 6 | **2.59** | **Yes** | 24 | 6 |
|  |  | **Control** | | 22 | 38 | 42 | 18 |  |  | 30 | 30 |
| **Imran-ul-Hassan 2021** | **0.405** | **Intervention** | | 8 | 27 | 24 | 11 | **6.32** | **Yes** | 24 | 11 |
|  |  | **Control** | | 2 | 33 | 22 | 13 |  |  | 5 | 30 |
| **Silva-Jr 2019** | **0.215** | **Intervention** | | 0 | 53 | 42 | 11 | **NA** | **NA** | 42 | 11 |
|  |  | **Control** | | 0 | 67 | 53 | 14 |  |  | 53 | 14 |
| **Spence 2020** | **0.59** | **Intervention** | | 55 | 334 | 192 | 197 | **0.78** | **No** | 192 | 197 |
|  |  | **Control** | | 72 | 339 | 211 | 200 |  |  | 211 | 200 |
| **Sultan 2010** | **0.405** | **Intervention** | | 22 | 28 | 39 | 11 | **2.22** | **Yes** | 39 | 11 |
|  |  | **Control** | | 40 | 113 | 107 | 46 |  |  | 70 | 83 |
| **Yu 2017** | **0.305** | **Intervention** | | 10 | 36 | 35 | 11 | **1.98** | **Yes** | 35 | 11 |
|  |  | **Control** | | 3 | 43 | 33 | 13 |  |  | 11 | 35 |
