# Supplementary material for: Assessing the robustness of meta-analyses to variability in the frequency and timing of postoperative delirium assessment – a novel methodological approach
Source: PLoS One. 2026 Oct 22;21(10):e0361124. doi: 10.1371/journal.pone.0361124 (PMC13690229; doi:10.1371/journal.pone.0361124)
Supplement: Supplementary file 3 [file pone.0361124.s004.docx]

Supporting Material

**S4:** Additional Analysis – Evaluating the Sensitivity of the Method to Expert-based Weights

Comparison of meta-analysis results between the original published studies, and the proposed sensitivity analysis conducted using weighted factor scores based on expert-based rankings and based on equal weights.

|  | **Observational studies** | | | **RCTs** | | |
| --- | --- | --- | --- | --- | --- | --- |
|  | **Point estimate** | **95% CI** | **Heterogeneity** | **Point estimate** | **95% CI** | **Heterogeneity** |
| **Original results) as reported in the published SRs** | 3.92 | 1.98-7.73 | 62%* | 1.19 | 0.89-1.57 | 77%* |
| **Primary sensitivity analysis (using factor weights based on expert-based ranking)** | 1.49 | 1.01-2.22 | 33% | 1.01 | 0.81-1.27 | 0% |
| **Supplementary analysis (using equal weights for all factors)** | 1.47 | 1.01-2.13 | 29% | 1.01 | 0.81-1.26 | 0% |
