# Supplementary material for: Assessing the robustness of meta-analyses to variability in the frequency and timing of postoperative delirium assessment – a novel methodological approach
Source: PLoS One. 2026 Oct 22;21(10):e0361124. doi: 10.1371/journal.pone.0361124 (PMC13690229; doi:10.1371/journal.pone.0361124)
Supplement: Supplementary file 4 [file pone.0361124.s005.docx]

Supporting Material

**S5:** Additional Analysis – Power Transformation Analysis

We performed supplementary analyses in which we used power transformation mapping for equation b, such as *(1 – confidence score)^K^ =* $\frac{False negative POD cases}{Reported negative POD cases}$ and used values for k to represent a conservative (k >1 – estimated false omission rate declines more steeply as confidence increases), or aggressive (k <1 – elevated false omission rates persist even at moderate to high confidence scores) scenarios. The original linear mapping corresponds to k = 1. We re-ran the analyses using k= 0.5 and k=2 and we report the results here.

Across this range of scenarios, the direction, overall point estimates and heterogeneity remained quite stable with changes in the pooled estimate, heterogeneity and precision consistent with our proposed sensitivity analysis using linear mapping. We have added a summary table below showing point estimates with 95% confidence intervals (CIs) and heterogeneity across different scenarios for the reviewer’s convenience.

|  | **Observational studies** | | | **RCTs** | | |
| --- | --- | --- | --- | --- | --- | --- |
|  | **Point estimate** | **95% CI** | **Heterogeneity** | **Point estimate** | **95% CI** | **Heterogeneity** |
| **Original results** (as reported in the published SRs) | 3.92 | 1.98-7.73 | 62%* | 1.19 | 0.89-1.57 | 77%* |
| **Linear mapping** (proposed approach) | 1.49 | 1.01-2.22 | 33% | 1.01 | 0.81-1.27 | 0% |
| **Non-linear mapping** (Aggressive scenario) | 1.29 | 0.92-1.81 | 0% | 1.01 | 0.79-1.31 | 0% |
| **Non-linear mapping** (Conservative scenario) | 1.77 | 1.11-2.81 | 51%* | 1.13 | 0.85-1.57 | 32% |
| *Significant heterogeneity | | | | | | |
