# Supplementary material for: Assessing the robustness of meta-analyses to variability in the frequency and timing of postoperative delirium assessment – a novel methodological approach
Source: PLoS One. 2026 Oct 22;21(10):e0361124. doi: 10.1371/journal.pone.0361124 (PMC13690229; doi:10.1371/journal.pone.0361124)
Supplement: Supplementary file 5 [file pone.0361124.s001.docx]

Supporting Material

**S1:** Details of the Survey Questionnaire for Expert Panelists
